# The National Pension Fund

**Published:** 1887-08-13

**Authors:** 


### The National Pension Fund.

It will save a good deal of trouble if nurses and other hospital officials who propose joining the National Pension Fund will send their names and other particulars to *the Secretary of The Hospitals Association*, Norfolk House, Norfolk Street, W.C., and not to the Editor of The Hospital. Names continue to arrive in considerable numbers, but if those interested desire the Fund to be started before the end of the Jubilee year they must influence and interest all their friends immediately.

### Asylum Nurses.

Much is written, and still more is thought, about the necessity for reform in connection with Asylum matters and Lunacy Laws. It is very right and proper that the subject should be thoroughly investigated and brought under the consideration of an intelligent reading public. There are abuses in connection with mental suffering that need rooting out of the apparent mystery and atmosphere of hopelessness that surround them. It is only as we deal with facts, as far as we are able to ascertain them, and fearlessly face the truth in relation to individual cases, our own family histories, and the various methods adopted in the treatment of patients while under asylum care, that we can really grapple with mental disease, and fortify ourselves against its inroads. But however earnest and uncompromising we may be in dealing with known or suspected evils, whether to be found in the experience of patients under asylum care, or often in the management of cases previous to their reception at such hands, we must be careful to avoid injustice.

One fact remains patent to the greatest enthusiast in the cause of "patients." There *must* be such homes for the insane of our population, after a certain stage of their disorder has been reached. Let us never fail to remember, that as the days go by in which we are yearning and striving, and if we would do true work, *praying* for our heart's desires in relation to lunacy reforms, our mental sufferers are receiving, even now, an amount of patience and forbearance at the hands of "strangers" which should call forth our heartiest approval and sincerest gratitude.

Much is being written in relation to a National Pension Fund for those engaged in nursing. It is especially needed with regard to "asylum nurses," who, from the peculiar nature of their work, have a double claim upon the consideration of intelligent people, whether in illness or old age, and who, if prevented by ill-health from continuing their employment, find it difficult, nay, almost impossible to find entrance to domestic service, or other spheres of labour, if their past experience is known. We rejoice that some effort is being made to induce workers in all kinds of hospitals to "lay by" for a "rainy day." May those of us who care truly for the welfare and spiritual, as well as temporal, happiness of those whom we have often proved "true friends," in their sphere and opportunity, help and cheer them on all we can in this needed direction of self-help. May we support them with our kind words, and aid them in every practical way in which, by a little self-denial, we can share the "care" and lighten the labours, and help guard "nurses" against the evils which undeniably attend, in a very special manner, work in our hospitals for the care and treatment, and, thank God, recovery of our mental sufferers.

From the Lady Superintendent of the Staffordshire Institution for Trained Nurses.

If the rules, when made, are such as they can agree to, I think the majority of the nurses here will like to belong to the National Pension Fund. There are fifty on the staff. The usual question, when I mention the Fund to them, is: Can I withdraw what I invest if unable to work, and will my friends, in case of my death, receive any or all I had paid in? I am afraid you will not make headway until the rules are made. I shall be glad to do all I can to help the scheme.

From a Private Nurse.

I am most anxious to join the National Pension Fund, and as soon as you have made your plans, I shall forward my money. I really think if a nurse has no family ties, and she gets £20 a year, she really ought to be able to pay £5 each year for her future. If she were ill she would have to pay much more to her friends for keep. I have long longed for such a Fund to rise up, for a nurse who belongs to a Nurses' Institute, when ill, except when she takes fever, has to leave the Home, and if she has no home, which is the case with me, what is to become of her? I doubt if the nurses can be grateful enough to the Editor of The Hospital for bringing this about. So I do hope we shall really be able to get this started.

From a Hospital Nurse.

I beg to bring before your notice one or two questions with regard to the Nurses' Pension Fund. They are as follows: If a person from fifty to sixty years of age who has been a nurse for twenty years of her life or more, has a sum of money which she is willing and able to invest in the funds—we will say £100—what annuity would be received from it: and would the proposition be accepted from such nurses at all at that age? I also enclose the names of my fellow-nurse and myself for your list of names, both trained nurses at the Eye and Ear Hospital, Bradford. We think the yearly sum to be paid by the nurses should be less than £5 per year, and should be returnable, at least in part, in cases of necessity.

